# Evaluation of wound healing activity of *Allamanda cathartica. L. *and *Laurus nobilis. L. *extracts on rats

**DOI:** 10.1186/1472-6882-6-12

**Published:** 2006-04-05

**Authors:** Shivananda Nayak, Poorna Nalabothu, Steve Sandiford, Vidyasagar Bhogadi, Andrew Adogwa

**Affiliations:** 1 Department of Pre clinical Sciences, Biochemistry unit, Faculty of Medical Sciences, The University of the West Indies, St. Augustine, Trinidad; 2 Veterinary school of Medicine, Faculty of Medical Sciences, The University of the West Indies, St. Augustine, Trinidad

## Abstract

**Background:**

*Allamanda cathartica. L. *is a perennial shrub used in traditional medicine for treating malaria and jaundice. *Laurus nobilis*. *L. *is a tree and has been used for its astringent, healing and diuretic properties. The objective of this study was to investigate the aqueous extracts of *Allamanda *and *Laurus nobilis *to evaluate their wound healing activity in rats.

**Methods:**

Excision and incision wound models were used to evaluate the wound healing activity of both the extracts on Sprague Dawley rats. In each model, animals were divided into four groups of 10 animals each. In both the model, group 1 served as control and group 2 as reference standard. In an excision wound model, group 3 animals were treated with *Allamanda *(150 mg kg^-1 ^day^-1^) and group 4 animals were treated with *Laurus nobilis *(200 mg kg^-1 ^b.w day^-1^) for 14 days respectively. In the case of incision wound model, group 3 and 4 animals were treated with the extracts of *Allamanda *and *Laurus respectively *for 10 days. The effects of vehicles on the rate of wound healing were assessed by the rate of wound closure, period of epithelialisation, tensile strength, weights of the granulation tissue, hydroxyproline content and histopathology of the granulation tissue.

**Results:**

The aqueous extract of *Allamanda *promoted wound healing activity significantly in both the wound models studied. High rate of wound contraction (P < .001), decrease in the period of epithelialisation (10.2 ± 0.13), high skin breaking strength (440.0 ± 4.53), significant increase in the weight of the granulation tissue (P < .001) and hydroxyproline (P < .001) content were observed in animals treated with the aqueous extract of *Allamanda*. Histological studies of the granulation tissue from the *Allamanda *treated group showed the presence of a lesser number of inflammatory cells, and increased collagen formation than the control. In *Laurus nobilis *treated animals, the rate of wound contraction, weight of the granulation tissue and hydroxyproline content were moderately high (P < .05). The histological study of the granulation tissue of the *Laurus nobilis *treated animals showed larger number of inflammatory cells, and lesser collagen when compared with the *Allamanda *treated group of animals. However, it was better than the control group of animals.

**Conclusion:**

The data of this study indicated that the leaf extract of *Allamanda *possesses better wound healing activity than the *Laurus nobilis *and it can be used to treat different types of wounds in human beings too.

## Background

Normal wound healing response begins the moment the tissue is injured. Wound healing is the process of repair that follows injury to the skin and other soft tissues. Following injury, an inflammatory response occurs and the cells below the dermis begin to increase collagen production. Later, the epithelial tissue is regenerated [1].

*Allamanda cathartica Linn*. (Apocyanaceae), a widely growing perennial shrub. The leaves are smooth and thick. In Suriname's traditional medicine, the roots are used against jaundice, complications with malaria and enlarged spleen. The flowers act as a laxative. Yellow *Allamanda *has also antibiotic action against Staphylococcus.

*Laurus nobilis Linn*. (Lauraceae) is a hardy evergreen tree that grows wild or cultivated. *Laurus nobilis *have been used for their astringent, carminative, diaphoretic, digestive, diuretic, emetic and stomachic properties. Bay Oil or Oil of Bays (Oleum Lauri) is used in liniments for bruising and sprains. It was once used to keep moths away, owing to its lauric acid content which gives it insecticidal properties. In the essential oil from the leaves, mainly monoterpenoides were found: Linalool (50%) is the major compound, whereas α-pinene, *p*-cymene, β-pinene and limonene range around 5 to 10% each. Phenylpropanoids appear only in traces: Newer work reports 1% cinnamic aldehyde and no eugenol, whereas older literature speaks of traces of both compounds.

The objectives of the pharmacology of wound healing are to study the influence of various measures in wound management programmes on healing and to screen drugs that promote healing. Several materials have so far been used and are reported to affect healing differently. However, intensive research in wound healing has not yielded, economic and efficacious pro-healing agent that could obviate the long hospitalization of patients following surgery and wound infliction. The present investigations were planned to evaluate the wound healing activity of *Allamanda cathartica *and *Laurus nobilis *extracts.

## Methods

### Plant material and preparation of the extract

Fresh leaves of *Allamanda cathartica *and *Laurus nobilis *were collected locally in June 2005 and identified by the Department of Botany, Life Sciences, The University of the West Indies, St. Augustine, Trinidad.

For the aqueous extract, 250 g each of *Allamanda cathartica and Laurus nobilis *were washed with water and air-dried, following which the leaves were ground into a paste using an electric blender and 50 ml of deionised water. The contents were filtered and the clear filtrate was used for the study. Both the extracts were subjected to preliminary phytochemical tests

### Animals

Healthy inbred Sprague Dawley rats of either sex, weighing between 200 g and 220 g were obtained from the animal house of the School of Veterinary Medicine, University of the West Indies. The rats were housed in polypropylene cages on normal food and water *ad libitum*. Animals were periodically weighed before and after experiments. The rats were anaesthetized prior to infliction of the experimental wounds. The surgical interventions were carried out under sterile conditions using ketamine anaesthesia (10 mg/kg). Animals were closely observed for any infection; those which showed signs of infection were separated and excluded from the study. An acute toxicity study was conducted for the extracts by the stair-case method [2]. The study was approved by the Ethics Committee of the Faculty of Medical Sciences, The University of the West Indies, St. Augustine

### Wound healing activity

Excision and incision wound models were used to evaluate the wound – healing activity of *Allamanda cathartica and Laurus nobilis*.

### Excision wound

The rats were inflicted with excision wounds as described by Morton and Malon [3]. The rats were anaesthetized prior to creation of the wounds, with 1 ml of intravenous ketamine hydrochloride (10 mg/kg body weight). The dorsal fur of the animal was shaved with an electric clipper and the area of the wound to be created was outlined on the back of the animals with methylene blue using a circular stainless steel stencil. A full thickness of the excision wound of 2.5 cm in width (circular area = 4.90 cm^2^) and 0.2 cm depth was created along the markings using toothed forceps, a surgical blade and pointed scissors. The entire wound left open [4,5]. The animals were divided into four groups of 10 each. The group 1 animals were left untreated and considered as the control. Group 2 animals served as reference standard and treated with sulphathiazole ointment. Animals of groups 3 and 4 were treated with aqueous extract of *Allamanda cathartica *(150 mg kg^-1 ^day^-1^) and *Laurus nobilis *(200 mg kg^-1 ^b.w day^-1^) respectively for 14 days. The parameters studied were wound closure, epithelialisation time, collagen content and weight of the tissue. The measurements of the wound areas of the excision wound model were taken on 1^st^, 5^th ^and 15^th ^day following the initial wound using transparent paper and a permanent marker. The recorded wound areas were measured with graph paper. The period of epithelialisation was calculated as the number of days required for falling of the dead tissue remnants without any residual raw wound.

In the excision wound model, granulation tissue formed on the wound was excised on the 15^th ^postoperative day and its weight recorded. The tissue was dried in an oven at 60°C and the dry weight was again noted. The protein content of the tissue extract was also estimated [6]. Acid hydrolysate of the dry tissue was used for the determination of hydroxyproline [7].

### Incision wound

The rats were anaesthetized prior to and during creation of the wounds, with 1 ml of intravenous ketamine hydrochloride (10 mg/kg body weight). The dorsal fur of the animals was shaved with an electric clipper. A longitudinal paravertebral incision of 6 cm long was made through the skin and cutaneous tissue on the back as described by Ehrlich and Hunt [8]. After the incision, the parted skin was sutured 1 cm apart using a surgical thread and curved needle. The wounds were left undressed. Extracts were topically applied to the wound once a day. The sutures were removed on 8^th ^post wound day and continued the application of the extract. The skin-breaking strength was measured by the method of Lee [9] on the 10^th ^day evening after the last application. Then the granulation tissue was taken on the 11^th ^day for further studies.

### Histopathological study

The healing tissues obtained on the 11^th ^day from all four groups of animals of the incision wound model were processed for histological study. The amount of collagen was quantified using Vangeison stain.

### Statistical analysis

Results, expressed as mean ± SE were evaluated using the t-test. Values of P < 0.001 were considered statistically significant.

## Results

In both the models studied, significantly improved wound-healing activity has been observed with the *Allamanda cathartica *leaf extract, compared to that of the reference standard and control group of animals. In the excision wound model, *Allamanda cathartica *treated animals showed significant reduction in the wound area (P < .001), faster rate of epithialisation (10.2 ± 0.13), increased dry weight of the tissue (P < .001) and increased hydroxyproline content (P < .001) when compared with the control group of animals. But the animals treated with *Laurus nobilis *extract showed moderate reduction in the wound area (P < .05) and slower rate of epithelialisation (11.7 ± 0.15). Both hydroxyproline and granulation tissue weight were moderately high (P < .05) in comparison to the control group of animals. Table 1 shows the wound area and other biochemical observations of all the four group of animals in excision wound model

**Table 1:** Effects of topical application of *Allamanda cathartica *and *Laurus nobilis *in excision wound model

Parameter	Control	Standard	*Allamanda *extract	*Laurus nobilis *extract
Wound area (mm^2^):				
Day 1	190.0 ± 1.83	190.0 ± 1.83	190.0 ± 1.83	190.0 ± 1.83
Day 5	151.3 ± 1.50	122.4 ± 4.14	125.7 ± 5.22	141.6 ± 5.6
Day 15	76.2 ± 1.24	42.5 ± 4.40	43.5 ± 2.1**	48.0 ± 4.33
Period of epithelialisation	14.1 ± 0.10	9.8 ± 0.13	10.2 ± 0.13**	11.7 ± 0.15
Hydroxyproline (mg g^-1^)	32.2 ± 2.11	57.1 ± 1.73	67.1 ± 7.39**	49.50 ± 4.60

Values are expressed as mean ± SE, n = 10 animals in each group P < .001 when compared to control

Table 2 depicts the wound healing effect of *Allamanda cathartica *in the incision wound model. In an incision wound model, *Allamanda cathartica*-treated animals demonstrated significant skin-breaking strength up to 440.0 ± 4.53 when compared to control animals (320.13 ± 3.23). Significant increase in the weight of the granulation tissue (P < .001) and hydroxyproline (P < .001) content were observed in animals treated with the aqueous extract of *Allamanda cathartica *when compared to the control group of animals. *Laurus nobilis *treated animals, showed moderate rate of wound closure, less skin-breaking strength (390.0 ± 3.40), moderately increased weight of the granulation tissue and hydroxyproline content (P < .05).

**Table 2:** Effects of topical application of *Allamanda cathartica *and *Laurus nobilis *in incision wound model

Parameter	Control	Standard	*Allamanda *extract	*Laurus nobilis *extract
Breaking strength (g)	320.13 ± 3.23	470.5 ± 4.1	440.0 ± 4.53**	390.0 ± 3.40
Granulation tissue wet weight ((mg)	87.1 ± 5.20	128.2 ± 4.20	125.7 ± 4.10**	107.3 ± 2.90
Granulation tissue dry weight ((mg)	13.0 ± 2.40	19.0 ± 0.68	20.0 ± 2.30 **	15.0 ± 0.48
Hydroxyproline (mg g^-1^)	173.6 ± 2.90	220.1 ± 3.32	216.1 ± 3.42**	183.6 ± 3.10

Values are expressed as mean ± SE, n = 10 animals in each group, P < .001 when compared to control

Toxicity studies showed that the maximum tolerated dose for the aqueous extract of *Allamanda cathartica *was 1.5 g/kg, b.w. and for the *Laurus nobilis *was 2 g/kg b.w. Therefore 150 mg/ kg, b.w. of *Allamanda *and 200 mg/kg, b.w. of *Laurus nobilis *extracts were selected for the topical evaluation of wound healing activity.

**Figure 1:**
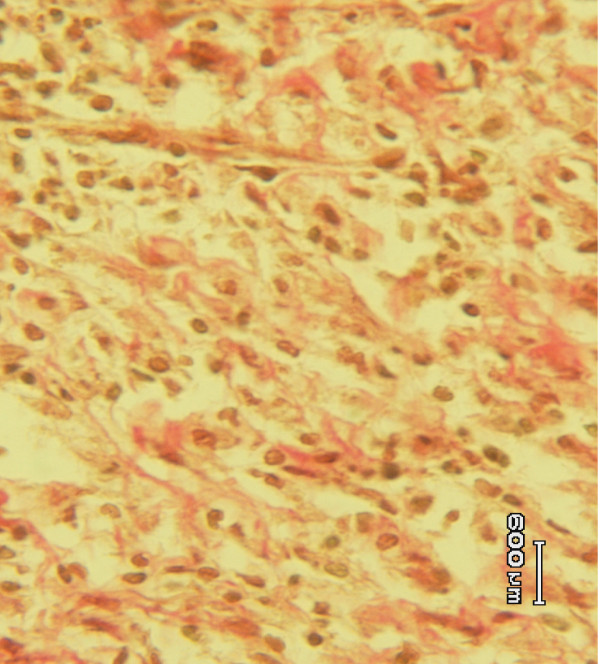
Granulation tissue of group 1 animal (control) showing with less collagen and more macrophages (Vangeison stain).

**Figure 2:**
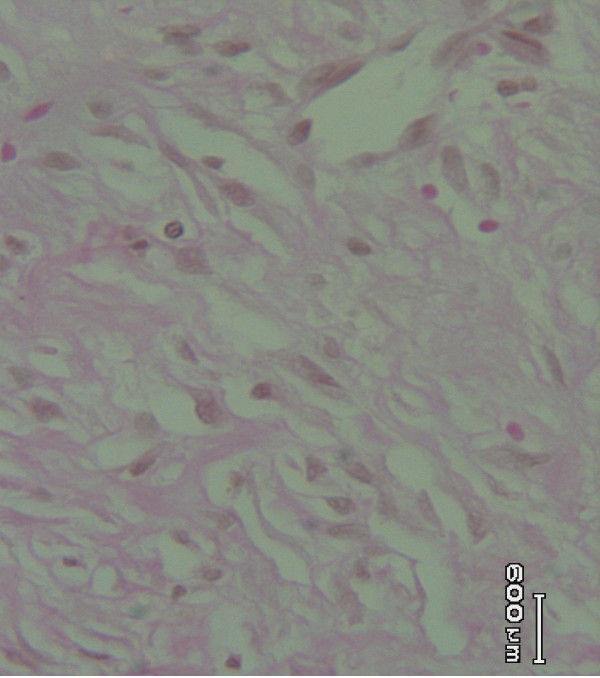
Granulation tissue of group 2 (standard) animal showing moderate deposition collagen (Vangeison stain).

**Figure 3:**
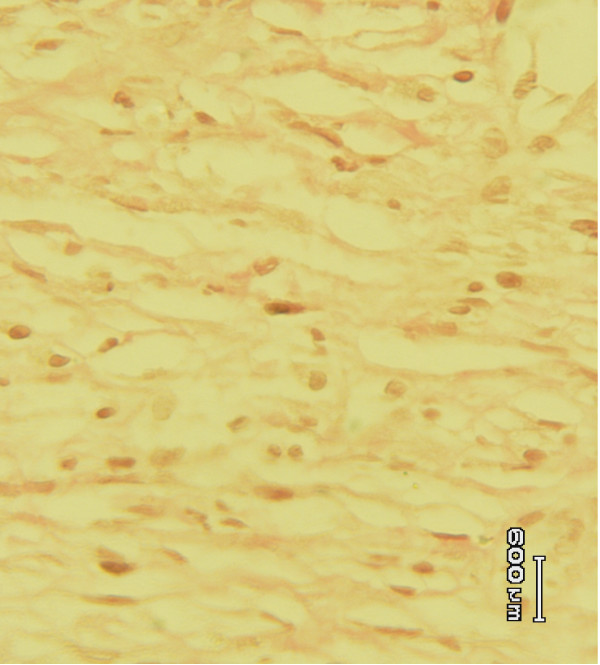
Granulation tissue of group 3 (*Allamanda *treated) animal showing more collagen and less macrophages (Vangeison stain).

**Figure 4:**
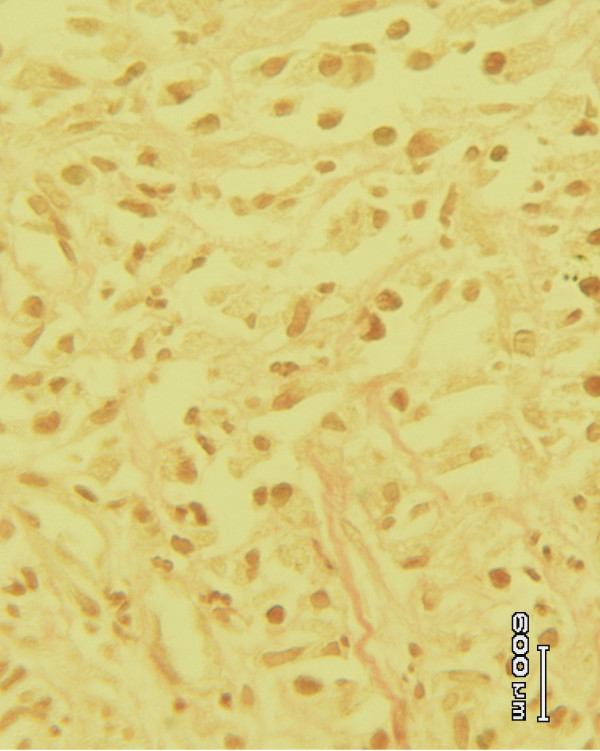
Granulation tissue of group 4 (*Laurus nobilis*) treated animal showing moderate deposition collagen (Vangeison stain).

Histological studies of the tissue obtained from the *Allamanda *treated (Figure 3) group showed significant increase in collagen deposition, few macrophages, tissue edema and more fibroblasts. It was more or less equal to the animals treated with sulphathiazole (Figure 2). In the case of *Laurus nobilis *treated (Figure 4) animal groups, moderate collagen deposition, macrophages, tissue edema and fibroblasts were observed. The histological studies of the granulation tissue of the control group of animals (Figure 1) showed more aggregation of macrophages with lesser collagen fiber. The wound healing was more significant in *Allamanda *treated group of animals.

## Discussion

Wound healing is a process by which a damaged tissue is restored as closely as possible to its normal state and wound contraction is the process of shrinkage of area of the wound. It mainly depends on the repairing ability of the tissue, type and extent of damage and general state of the health of the tissue. The granulation tissue of the wound is primarily composed of fibroblast, collagen, edema, and small new blood vessels. The undifferentiated mesenchymal cells of the wound margin modulate themselves into fibroblast, which start migrating into the wound gap along with the fibrin strands. The collagen composed of amino acid (hydroxyproline) is the major component of extra cellular tissue, which gives strength and support. Breakdown of collagen liberates free hydroxyproline and its peptides. Measurement of the hydroxyproline could be used as an index for collagen turnover. The data depicted in table 1 showed that the hydroxyproline content of the granulation tissue of the animals treated with aqueous extract of *Allamanda cathartica *was significantly increased when compared to the control and the group of animals treated with *Laurus nobilis *indicating increased collagen turnover. In addition, increase in dry tissue weight also indicated the presence of higher protein content. The preliminary phytochemical analysis of aqueous leaf extract of *Allamnda cathartica *revealed the presence of flavanoids and triterpenoids whereas, *Laurus nobilis *extract showed the presence of alkaloids and monoterpenoids. Flavonoids are known to reduce lipid peroxidation not only by preventing or slowing the onset of cell necrosis but also by improving vascularity. Hence, any drug that inhibits lipid peroxidation is believed to increase the viability of collagen fibrils by increasing the strength of collagen fibres, increasing the circulation, preventing the cell damage and by promoting the DNA synthesis [10]. Flavonoids, [11] triterpenoids [12] are also known to promote the wound-healing process mainly due to their astringent and antimicrobial property, which seems to be responsible for wound contraction and increased rate of epithelialisation. Similar types of wound-healing activity were reported on *Vernonia arborea *[13] and *Pentas lanceolata *[14]. In the authors laboratory all the surgical interventions were carried out under sterile conditions and animals were closely observed for any infection; those which showed signs of infection were separated and excluded from the study. This is very important and rresearchers proved that the control microbial infection is necessary for better wound healing and its management [15,16].

Thus, wound-healing property of *Allamanda cathartica *and *Laurus nobilis *may be attributed to the phytoconstituents present in it, which may be either due to their individual or additive effect that fastens the process of wound healing. Between the two extracts studied, the *Allamanda cathartica *leaf extract was found to possess better wound-healing property. At this stage, it is difficult to say which component(s) of the extracts are responsible for this wound healing activity. However, further phytochemical studies are needed to isolate the active compound(s) responsible for these pharmacological activities.

## Conclusion

The leaf extracts of *Allamanda cathartica *and *Laurus nobilis *promote wound healing activity. The *Allamanda cathartica *extract showed remarkable wound healing activity and it may be suggested for treating various types' wounds in human beings. Further studies with purified constituents are needed to understand the complete mechanism of wound healing activity of *Allamanda cathartica*.

## Competing interests

The author(s) declare that they have no competing interests.

## Authors' contributions

SN was responsible for practically carrying out the experiments

PL, SSA and VSB were responsible for the preparation of plant extracts and phytochemical study

AA – contributed histological work

## Pre-publication history

The pre-publication history for this paper can be accessed here:


